# Relationship between post-stroke dysphagia and pharyngeal sensory impairment

**DOI:** 10.1186/s42466-023-00233-z

**Published:** 2023-02-16

**Authors:** Bendix Labeit, Anne Jung, Sigrid Ahring, Stephan Oelenberg, Paul Muhle, Malte Roderigo, Fiona Wenninger, Jonas von Itter, Inga Claus, Tobias Warnecke, Rainer Dziewas, Sonja Suntrup-Krueger

**Affiliations:** 1 Department of Neurology with Institute of Translational Neurology, University Hospital Muenster, Albert-Schweitzer-Campus 1; Building A1, 48149 Muenster, Germany; 2 Institute for Biomagnetism and Biosignal Analysis, University Hospital Muenster, Muenster, Germany; 3 Department of Neurology and Neurorehabilitation, Klinikum Osnabrueck – Academic teaching hospital of the WWU, Muenster, Germany

**Keywords:** Stroke, Oropharyngeal dysphagia, Post stroke dysphagia, Aspiration, Pneumonia

## Abstract

**Background:**

Post-stroke dysphagia (PSD) is common and can lead to serious complications. Pharyngeal sensory impairment is assumed to contribute to PSD. The aim of this study was to investigate the relationship between PSD and pharyngeal hypesthesia and to compare different assessment methods for pharyngeal sensation.

**Methods:**

In this prospective observational study, fifty-seven stroke patients were examined in the acute stage of the disease using Flexible Endoscopic Evaluation of Swallowing (FEES). The Fiberoptic Endoscopic Dysphagia Severity Scale (FEDSS) and impaired secretion management according to the Murray-Secretion Scale were determined, as well as premature bolus spillage, pharyngeal residue and delayed or absent swallowing reflex. A multimodal sensory assessment was performed, including touch-technique and a previously established FEES-based swallowing provocation test with different volumes of liquid to determine the latency of swallowing response (FEES-LSR-Test). Predictors of FEDSS, Murray-Secretion Scale, premature bolus spillage, pharyngeal residue, and delayed or absent swallowing reflex were examined with ordinal logistic regression analyses.

**Results:**

Sensory impairment using the touch-technique and the FEES-LSR-Test were independent predictors of higher FEDSS, Murray-Secretion Scale, and delayed or absent swallowing reflex. Decreased sensitivity according to the touch-technique correlated with the FEES-LSR-Test at 0.3 ml and 0.4 ml, but not at 0.2 ml and 0.5 ml trigger volumes.

**Conclusions:**

Pharyngeal hypesthesia is a crucial factor in the development of PSD, leading to impaired secretion management and delayed or absent swallowing reflex. It can be investigated using both the touch-technique and the FEES-LSR-Test. In the latter procedure, trigger volumes of 0.4 ml are particularly suitable.

**Supplementary Information:**

The online version contains supplementary material available at 10.1186/s42466-023-00233-z.

## Introduction:

Dysphagia is a common symptom after stroke and occurs in up to 80% of patients in the acute phase of the disease [1]. In addition to affecting quality of life [2] and placing a significant financial burden on the health care system [3] post-stroke dysphagia (PSD) causes serious complications such as malnutrition [4] and pneumonia [5], resulting in increased mortality [6].

Instrumental procedures such as the Videofluoroscopic swallowing study (VFSS) or Flexible Endoscopic Evaluation of Swallowing (FEES) have been developed to visualize swallowing. FEES and VFSS have comparable diagnostic metrics in the detection of relevant dysphagia pathologies and are therefore both considered as diagnostic gold standards [7]. In addition to reliable detection of PSD, instrumental procedures also allow characterization of the dysphagia phenotype, i.e., the pattern of dysphagia impairment [8]. However, the use in clinical routine is determined by methodological advantages and disadvantages of both modalities. In stroke patients, FEES has the distinct advantage that the examination can be performed at the bedside even in patients with limited ability to cooperate. Further, secretion management can be assessed.

In recent decades, an extensive cortical and subcortical network involved in the central control of swallowing has increasingly come into scientific focus. Studies using voxel-based lesion symptom mapping have demonstrated that post-central lesions, i.e. the primary sensory cortex, in particular are associated with severe forms of swallowing impairment [9]. In addition, individual studies have shown a correlation between sensory pharyngeal dysfunction and aspiration [10] or PSD severity [11]. This points to a specific role of the central sensory system in the pathophysiology of PSD and suggests that the sensory system exerts a secondary modulatory effect on swallowing motor function.

In clinical practice, it is difficult to determine the extent of pharyngeal sensory dysfunction. Sensory testing is mostly conducted, if at all, using qualitative or (semi-)quantitative methods such as the FEES-based touch-technique [12]. This involves touching structures of the pharynx and larynx with the endoscope and subjectively evaluating the patient’s response. However, a quantitative tool would be favorable for accurate assessment in disease progression and for elaborate analysis in scientific studies. Therefore, a FEES-guided swallowing provocation test was developed previously, in which different volumes of liquid are administered via a tube placed in the upper third of the oropharynx and the latency of swallowing response (LSR) is determined (FEES-LSR-Test). This test was validated in healthy subjects and clearly distinguished between the physiological state and experimentally induced pharyngeal anesthesia [1]. Further, prolonged LSR was the only clinical determinant of swallowing alterations in terms of presbyphagia in a cohort of healthy subjects older than 70 years [13]. However, the FEES-LSR-Test has not yet been used and validated in stroke patients.

The aims of this study were therefore (1) to evaluate the relationship between sensory impairment and dysphagia severity in acute stroke patients, (2) to investigate which dysphagia pathologies and phenotypes are linked to sensory impairment, (3) To verify the applicability/suitability of the FEES-LSR-Test in acute stroke patients compared the conventional touch-technique, and (4) to determine the ideal test volume in the LSR procedure to recommend an abbreviated but equally valid protocol in clinical practice. To this end, a prospective FEES study was conducted in a cohort of patients with acute stroke that included sensory evaluation using the touch-technique and the FEES-LSR-Test.

## Methods

### Patient cohort

Patients with acute stroke admitted to the intensive care unit (ICU) or stroke unit at University Hospital Münster between 12/2021 and 02/2022, in whom FEES was indicated according to our in-house standard (i.e., failure of swallowing screening test or symptoms predictive of dysphagia e.g., severe dysarthria, aphasia, National Institute of Health Stroke Scale [NIHSS] > 9) were prospectively included in the study. Patients were excluded if prior strokes or other premorbid conditions associated with dysphagia were known. As part of the review process, the number of patients that dropped out before study incusion were retrospectively recored and illustrated in a flow-diagram. All patients underwent FEES in the acute stage of disease including two different sensory test procedures according to the protocols described below. The following clinical data were recorded during acute hospitalization: type of stroke, aetiology of stroke, stroke severity (National Institute of Health Stroke Scale [NIHSS], Modified Rankin Scale [mRS]), therapeutic interventions, lesion location, intensive care interventions (intubation and tracheotomy), complications (pneumonia, death), and length of stay in the ICU/stroke unit and in the hospital overall. The study design was approved by the local ethics committee.

### Dysphagia assessment

The severity of dysphagia was determined using the Fiberoptic Endoscopic Dysphagia Severity Scale (FEDSS) ranging from 1 (no relevant dysphagia) to 6 (severe dysphagia with impaired secretion management), which is described in detail elsewhere[14].

Furthermore, additional information regarding the salient findings of dysphagia, i.e. premature bolus spillage, pharyngeal residue, and delayed or absent swallowing reflex, were collected. Premature bolus spillage was assessed according to the following ordinal scale: 0: no premature bolus spillage; 1: premature bolus spillage into the valleculae, 2: premature bolus spillage into the piriform sinus, and 3: premature bolus spillage with overflow into the laryngeal vestibule. Pharyngeal residue in the valleculae or in the piriform sinus were graded according to the following ordinal scale, based on the Yale Pharyngeal Residue Severity Scale [15]: 0: none or trace, 1: mild or moderate,2: severe. Attenuation or absence of the swallowing reflex was scored according to the following ordinal scale: 0: timely swallowing reflex with triggering of the reflex within less than 3 s after the bolus has reached the valleculae, 1: delayed swallowing reflex with triggering of the reflex after 3 s; 2: absent swallowing reflex.

Also, the functional oral intake scale [16] and the presence of a nasogastric feeding- or PEG tube at the time of discharge were recorded to assess functional recovery during the acute stage.

FEES was performed by two speech-language pathologists who had more than 5 years of experience in diagnostics of PSD and were both FEES certified according to the program of the European Society for Swallowing Disorders [17].

### Pharyngeal sensory assessment

Two different FEES-based methods were used to assess pharyngeal sensitivity, both of which have been published and validated. They have demonstrated good interrater reliability and are described in detail elsewhere [11, 18].

One procedure applies the conventional touch-technique. Here, different laryngeal structures on both sides of the pharynx were gently touched with the tip of the endoscope (the pharyngeal sidewalls, the pharyngeal posterior walls, and the arytenoids). Then, for each of the structures touched, the patient's response was scored: 0: normal: immediate swallow, cough, or laryngeal adductor reflex; 1: reduced: weak or delayed response; or 2: absent: no response. Thus, the sum-score for pharyngeal sensory impairment ranked from 0 to 12 points [11]. In assessing the semiquantitative outcome for each laryngeal structure, both examiners had to come to a consensus directly at the bedside.

The other method is the FEES-LSR-Test, in which the LSR is measured as an indicator of sensory impairment. Different volumes of liquid (0.2 ml, 0.3 ml, 0.4 ml, and 0.5 ml, 3 trials per unit volume each) were administered as swallow triggers via a nasal tube placed in the upper third of the oropharynx under FEES control. Subsequently, the time in seconds until the triggering of the swallowing reflex (or another protective reflex such as coughing or clearing the throat) was measured and the average time per volume unit was calculated. LSR was assessed after the examination using the recorded FEES video by an independent person who was blinded to the other FEES results and the results of the touch-technique.

### Statistical analysis

#### Comparison of the two sensory assessment methods

In a first step, the two methods of pharyngeal sensory assessment (touch-technique and FEES-LSR-Test) were compared. The purpose of this was twofold. First, to investigate whether both methods give equivalent results and thus are paradigms that represent the same condition. Second, to determine the optimal volume for the FEES-LSR-Test to investigate pharyngeal hypesthesia in stroke patients. Spearman's rank correlation coefficient was used to correlate the sum score of the touch-technique with the average LSR of each volume tested.

#### Relationship between pharyngeal sensory impairment and dysphagia

The relationship between pharyngeal sensory impairment according to both assessment methods and the severity of dysphagia according to the FEDSS, impairment of secretion management using the Murray Secretion Scale, premature bolus spillage, pharyngeal residue and delayed swallowing reflex was examined. In the analysis using the results of the FEES-LSR-Test, only the volumes that had shown a significant correlation with the touch-technique were examined (LSR at 0.3 ml and 0.4 ml). In each case, ordinal regression analysis was performed to predict the severity of dysphagia using the FEDSS, the extent of impaired secretion management using the Murray Secretion Scale, premature bolus spillage, pharyngeal residue, and delayed swallowing reflex using the ordinal ranking defined above. Independent variables used in each model were age, sex, stroke severity (NIHSS), and sensory impairment (touch-technique, LSR at 0.3 ml, and LSR at 0.4 ml, each in a separate regression analysis). The test of parallel lines was used to determine whether the proportional odds precondition was met. In cases where sensory impairment was a significant predictor, boxplots were created to illustrate the relationship by plotting sensory impairment data for each ordinal scale level.

All statistical analyses were done with IBM SPSS Statistics version 28. In case of missing data, the respective case was excluded from the analysis.

## Results

### Description of the patient cohort

**Fig. 1:**
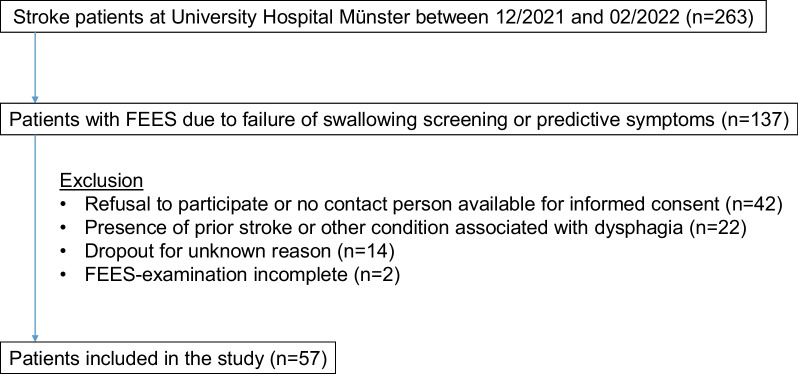
Flow-diagram for the exclision of patients

A total of 57 patients could be included in the study within the 3-months study period (a flow-diagram for the exclusion of patients is illustrated in Fig. 1). All dysphagia severity levels were represented, with approximately 20% of patients having no dysphagia and up to approximately 30% having severe dysphagia with penetration or aspiration of saliva. The demographic and clinical data of the patient cohort are illustrated in Table 1 and the results of the sensory testing, severity and characteristics of PSD are shown in Table 2. Most patients had severe dysphagia with aspiration of saliva (28%) followed by patients without dysphagia (23%). Otherwise, all FEDSS levels were represented in the cohort, with an FEDSS of 5 occurring in only 2 patients.

**Table 1:** clinical data of the patient cohort

*Demographics*	
Mean age in years ± SD	71.9 ± 12.4
Men/women n(%)	35 (61.4%)/22 (38.6%)
*Type of stroke, n (%)*	
Ischemia	54 (94.7%)
Hemorrhage	2 (3.5%)
Both	1 (1.8%)
*Etiology, ischemic stroke, n (%)*	
Large artery atherosclerosis	13 (22.8%)
Cardioembolic	18 (31.6%)
Other etiology	2 (3.5%)
Unknown	22 (38.6%)
*Etiology, hemorrhagic stroke, n (%)*	
Hypertension	2 (3.5%)
Other etiology	1 (1.8%)
*Stroke severity*	
NIHSS, mean ± SD	12.6 ± 5.9
mRS at discharge, mean ± SD	3.6 ± 1.4
*Interventions, n (%)*	
i.v. Thrombolysis	20 (35.1%)
Thrombectomy	33 (57.9%)
Craniotomy	4 (7.0%)
External ventricular drain	4 (7.0%)
*Lesion location, n (%)*	
Supratentorial	51 (89.5%)
Infratentorial	4 (7.0%)
Multiple locations	2 (3.5%)
*Lesion side n (%)*	
Left	28 (49.1%)
Right	24 (42.1%)
Both	5 (8.8%)
*Intensive care treatment, n (%)*	
Intubation	39 (68.4%)
Tracheotomy	2 (3.5%)
*Complications n (%)*	
Pneumonia	22 (38.5%)
Death	3 (5.3%)
*Mean length of stay in days ± SD*	
Stroke unit/ICU	7.5 ± 9.3
Total hospital	16.8 ± 16.1

*SD* standard deviation; *NIHSS* National Institute of Health Stroke Scale, *mRS* Modified Rankin Scale, *i.v.* intravenous; *i.a.* intra-arterial; *ICU* intensive care unit

**Table 2:** Results of the sensory testing and severity and characterization of dysphagia

*Sensory testing*	
Mean score touch-technique ± SD	4.1 ± 4.0
Mean LSR in s at 0.2 ml ± SD	2.5 ± 0.7
Mean LSR in s at 0.3 ml ± SD	2.4 ± 0.6
Mean LSR in s at 0.4 ml ± SD	2.1 ± 0.7
Mean LSR in s at 0.5 ml ± SD	1.9 ± 0.8
*Dysphagia severity*	
FEDSS 1, n (%)	13 (22.8%)
FEDSS 2, n (%)	7 (12.3%)
FEDSS 3, n (%)	12 (21.1%)
FEDSS 4, n (%)	7 (12.3%)
FEDSS 5, n (%)	2 (3.5%)
FEDSS 6, n (%)	16 (28.1%)
Murray 0, n (%)	22 (38.6%)
Murray 1, n (%)	19 (33.3%)
Murray 2, n (%)	7 (12.3%)
Murray 3, n (%)	9 (15.8%)
mean FOIS at discharge ± SD	4.5 ± 1.9
NGT/PEG at discharge, n (%)	13 (22.8%)
*Dysphagia characteristics, n (%)*	
No premature bolus spillage	9 (15.8%)
Premature bolus spillage valleculae	10 (17.5%)
Premature bolus spillage piriform sinus	31 (54.4%)
Premature bolus spillage laryngeal vestibule	6 (10.5%)
No or trace pharyngeal residue	12 (21.1%)
Mild or moderate pharyngeal residue	31 (54.4%)
Sever pharyngeal residue	12 (21.1%)
Normal triggering of swallowing reflex	26 (45.6%)
Delayed triggering of swallowing reflex	13 (22.8%)
Absent swallowing reflex	18 (31.6%)

*SD* standard deviation; *LSR* latency of swallowing response; *FEDSS* Fiberoptic Endoscopic Dysphagia Severity Scale; *FOIS* Functional Oral Intake Scale; *NGT/PEG* nasogastric tube or percutaneous endoscopic gastrostomy

### Correlation between the sensory assessment procedures

At 0.3 ml (*p* = 0.017; correlation coefficient: 0.33) and 0.4 ml (*p* = 0.001; correlation coefficient: 0.49), there was a significant correlation between the score of the touch-technique and the FEES-LSR-Test. However, for the other volumes, there was no correlation between the two different examination methods (0.2 ml: *p* = 0.120, correlation coefficient: 0.22; 0.5 ml: *p* = 0.076, correlation coefficient: 0.28). Therefore, the LSRs at the volumes of 0.3 ml and 0.4 ml were used for further analyses.

### Relationship between sensory impairment and dysphagia

**Fig. 2:**
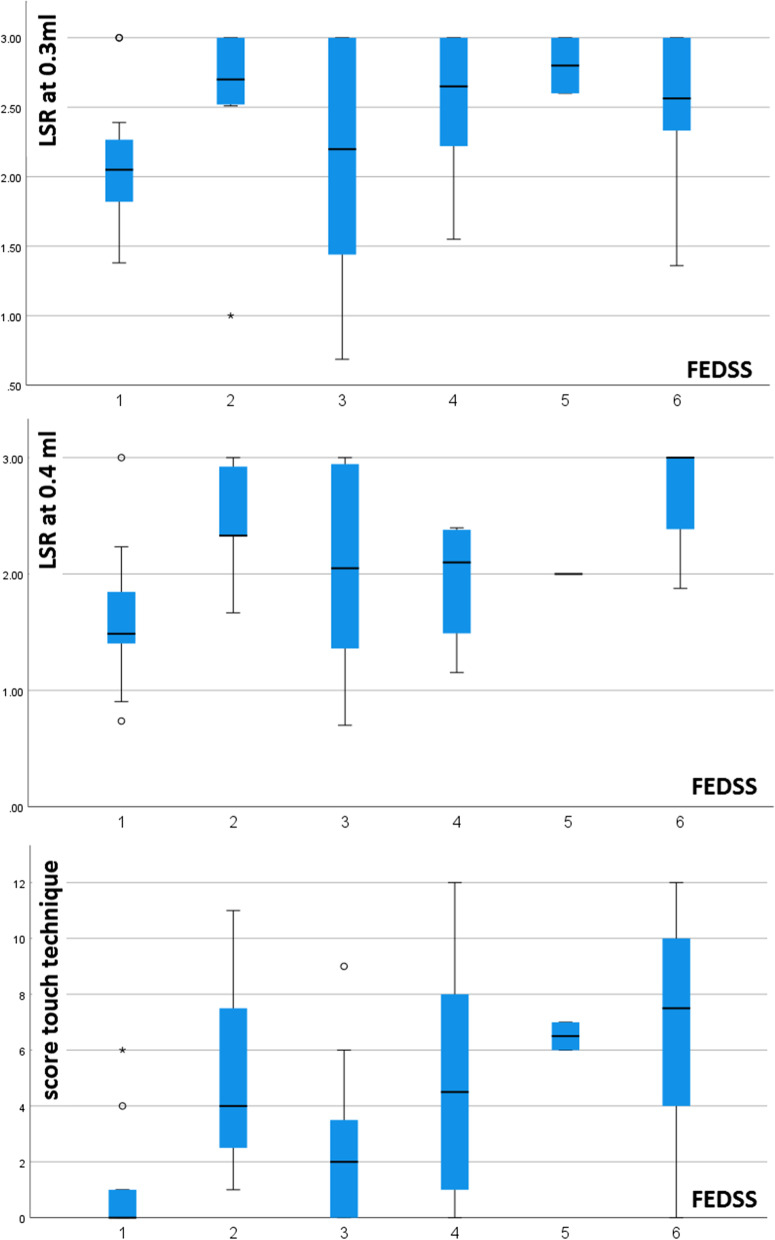
Distribution of latency of swallowing response (LSR) or touch-sum score for each Fiberoptic Endoscopic Dysphagia Severity Scale (FEDSS) level visualized in boxplots

**Fig. 3:**
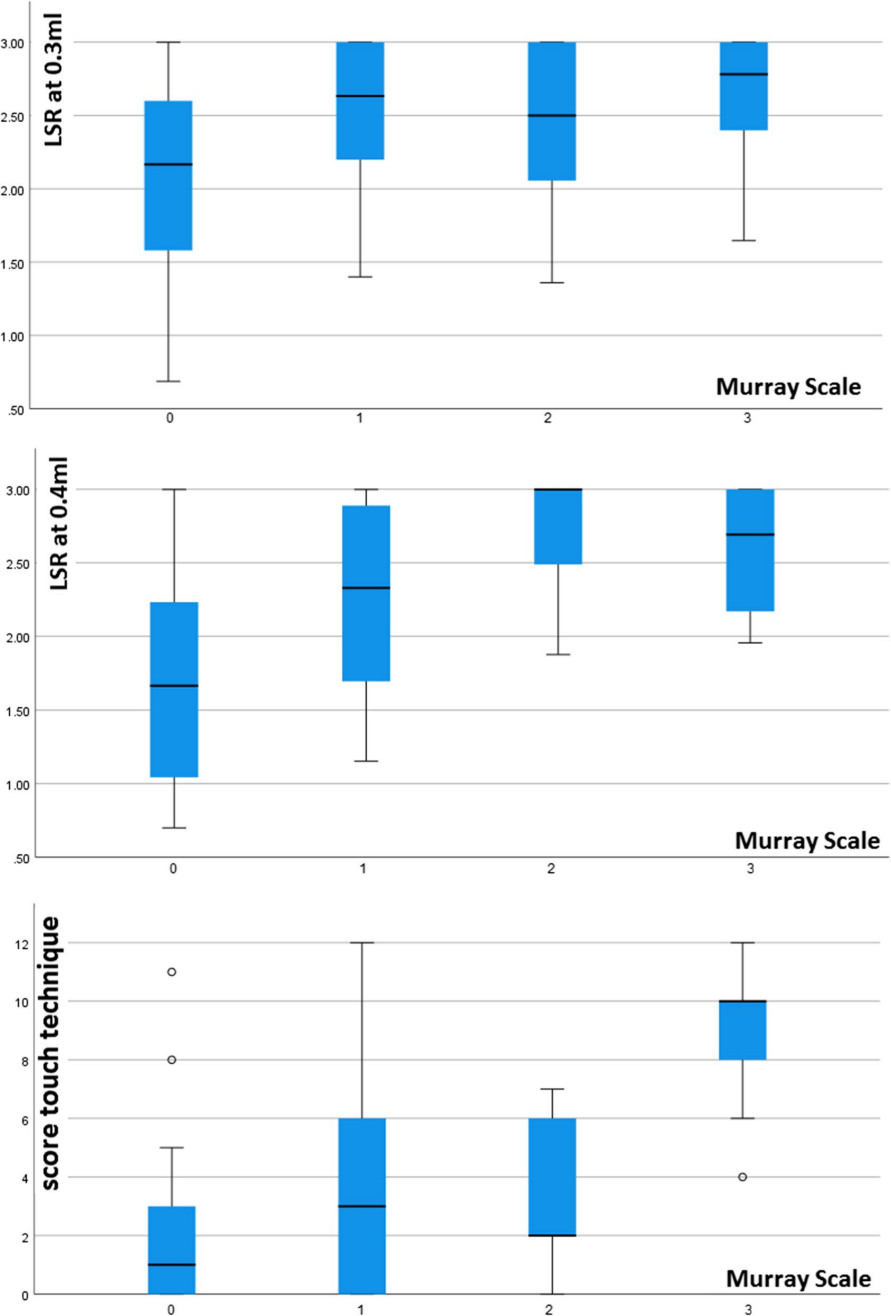
Distribution of latency of swallowing response (LSR) or touch-sum score for each Murray Secretion Scale level visualized in boxplots

**Fig. 4:**
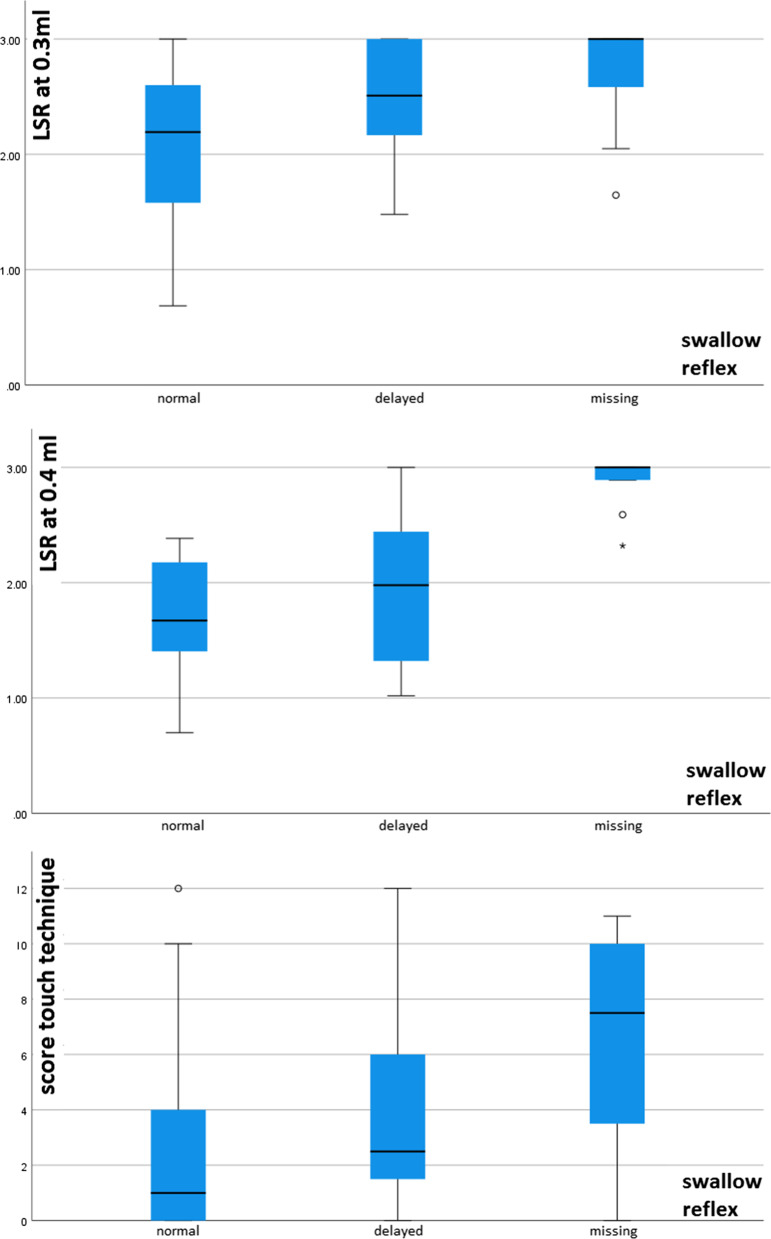
Distribution of latency of swallowing response (LSR) or touch-sum score for each swallowing reflex alteration level visualized in boxplots

In the regression analysis predicting severity of dysphagia, LSR at 0.4 ml and the touch-technique sum-score along with NIHSS were independent predictors of higher FEDSS. Figure 2 visualizes the distribution of LSR or touch-sum score for each FEDSS level in boxplots. When the Murray secretion scale was considered, all sensory measurement methods were independent predictors of impaired secretion management. Furthermore, in the regression analysis using LSR at 0.3 ml, NIHSS and male sex were additionally associated with increased Murray secretion scale. Figure 3 visualizes the distribution of the LSR or touch-sum-socre for each levels of the Murray Secretion Scale. In contrast to secretion impairment, none of the sensory tests were predictors of increased premature bolus spillage. Here, only increased NIHSS was a corresponding risk factor. Similarly, no predictors were identified in the analysis for pharyngeal residue, as no significant regression model was found. When considering an impaired swallowing reflex, all sensory test procedures were independent predictors, with the parallel lines condition being met only in the regression with touch technique. In addition to sensory deficits, older age was also shown to predispose to an impaired swallowing reflex in this analysis. Figure 4 visualizes the distribution of LSR or touch-sum-score for each level of swallowing reflex alteration. The model fitting information of the regression analyses and the results of the parallel line tests are shown in Additional file 1: Table S1. Table 3 shows the parameter estimates of the predictors analyzed in each of the analyses with a significant regression model.

**Table 3:** Parameter estimates of the predictors for the different regression analyses with a significant regression model

	FEDSS		Murray secretion scale		Premature bolus spillage		Impaired swallowing reflex	
	Odds ratio [95% CI]	*p*-Value	Odds ratio [95% CI]	*p*-Value	Odds ratio [95% CI]	*p*-Value	Odds ratio [95% CI]	*p*-Value
*LSR at 0.3 ml*								
Age	1.008 [0.966–1.052]	0.713	1.034 [0.989–1.080]	0.138	1.015 [0.972–1.061]	0.496	1.049 [0.996–1.103]	0.068
Men	2.709 [0.970 -7.561]	0.057	3.004 [0.998–9.046]	0.050*	2.138 [0.691–6.616]	0.187	2.218 [0.683–7.200]	0.185
NIHSS	1.199 [1.089–1.321]	< 0.001*	1.115 [1.018–1.222]	0.019*	1.142 [1.036–1.260]	0.008*	1.053 [0.953–1.164]	0.308
LSR	1.916 [0.822–4.469]	0.132	2.654 [1.083–6.506	0.033*	1.857 [0.777–4.441]	0.164	5.618 [1.886–16.737]	0.002*
*LSR at 0.4 ml*								
Age	0.971 [0.925–1.308]	0.237	1.001 [0.953–1.052]	0.959	1.009 [0.960–1.060]	0.718	1.033 [0.974–1.096]	0.282
Men	2.317 [0.745–7.205]	0.147	1.801 [0.537–6.036]	0.34	1.916 [0.544–6.752]	0.311	2.242 [0.573–8.776]	0.246
NIHSS	1.175 [1.055–1.308]	0.003*	1.071 [0.968–1.184]	0.184	1.145 [1.020–1.284]	0.021*	1.066 [0.948–1.198]	0.285
LSR	3.535 [1.402–8.912]	0.007*	4.322 [1.645–11.359]	0.003*	2.000 [0.802–4.985]	0.137	10.541 [3.195–34.782]	< 0.001*
*Touch*								
Age	0.968 [0.918–1.021]	0.229	0.994 [0.994–1.047]	0.821	0.998 [0.946–1.052]	0.929	1.077 [1.014–1.145]	0.017*
Men	1.634 [0.531–5.031]	0.392	1.536 [0.462–5.108]	0.484	1.252 [0.373–4.196]	0.716	2.689 [0.736–9.818]	0.134
NIHSS	1.159 [1.042–1.290]	0.007*	1.081 [0.980–1.193]	0.12	1.132 [1.018–1.260]	0.022*	1.059 [0.947–1.184]	0.314
Touch	1.347 [1.134–1.600	< 0.001*	1.324 [1.120–1.565]	< 0.001*	1.107 [0.938–1.307]	0.229	1.227 [1.046–1.440]	0.012*

*FEDSS* Fiberoptic Endoscopic Dysphagia Severity Scale; *CI* Confidence interval; *NIHSS* National Institute of Health Stroke Scale; *LSR* Latency of Swallowing Response; * and bold printing indicate statistically significant *p*-values

## Discussion

The main finding of our study was that pharyngeal hypesthesia is an independent predictor of the severity of PSD and impaired secretion management. In line with this finding, previous studies have shown an association between sensory deficits and dysphagia as well [10, 11, 19, 20, 21]. In contrast to these previous studies, the present study has now succeeded in demonstrating that this association persisted after accounting for other relevant cofactors such as stroke severity. Thus, the results suggest that pharyngeal hypesthesia is a relevant factor in the development of PSD.

With regards to patterns of swallowing impairment, we found an association between pharyngeal hypesthesia and a delayed or absent swallowing reflex. However, there was no association with the extent of pharyngeal residue or premature bolus spillage. This suggests that the crucial PSD mechanism caused by sensory impairment is mediated by an absent or delayed swallowing reflex. Evidence for other secondary motor modulatory effects induced by pharyngeal hypesthesia leading to premature bolus spillage or pharyngeal residue was not found in our study. In contrast, Sulica et al. reported an increase in premature bolus spillage and pharyngeal residue caused by pharyngeal anesthesia in healthy participants [22]. However, in stroke patients decreased pharyngeal contractility, attenuated tongue retraction, hypercontractility of the upper esophageal sphincter, bulbar and pseudobulbar oral paresis, and deficits in higher cortical functions may play a more vital role in the development of pharyngeal residue and premature bolus spillage. A dysfunctional swallowing reflex seems to affect not only the severity of dysphagia in general, but also the management of secretions in particular. One plausible reason for this could be that the pooling of secretions due to pharyngeal hypesthesia is not sufficiently perceived by the patient and thus does not trigger swallowing. This may result in a risk of saliva aspiration.

The underlying mechanisms of pharyngeal hypesthesia in stroke seem to be heterogeneous and are not well understood in detail. On the one hand, central mechanisms of sensory impairment are a major contributing factor. This is illustrated by the association of particularly severe dysphagia with lesions in the post-central cortex as the primary sensory area [9]. Electrophysiological study results also provided evidence of central hypesthesia in PSD. In contrast to stroke patients without dysphagia, patients with PSD showed reduced ipsilesional activation of sensory evoked potentials to pharyngeal stimulation [23]. Also, abnormal asymmetric sensory evoked potentials were described in the majority of patients during pharyngeal electrical stimulation [24]. Furthermore, contralesionally decreased pharyngeal sensory evoked potentials were associated with increased duration of swallowing [25]. In addition to these central effects, peripheral mechanisms of pharyngeal hypesthesia have also been postulated. This includes substance P, a neuropeptide that is released into saliva by sensory nerve endings. It is assumed that the degeneration of these peripheral sensory nerves results in a decrease of substance P, which mediates dysphagia [26]. In stroke patients, a decreased salivary substance P level was found to be a predictor of reduced swallowing frequency, independent of age, stroke severity, and vigilance. In addition, a low substance P level was associated with a higher rate of pneumonia [27]. Possible reasons for peripheral deficits may include mucosal damage, e.g., due to interventions such as intubation or tube placement. In summary, studies suggest that perception and integration of sensory information is impaired at both central and peripheral levels, although the complex interactions are not yet understood in detail.

Therapeutic approaches targeting the sensory system with neurostimulation have shown promising study results. One method is pharyngeal electrical stimulation (PES), in which a sensory stimulus is used with the aim to trigger central neuroplasticity. In a multicenter randomized controlled trial, pharyngeal electrical stimulation increased decannulation rates in stroke patients by improving PSD and secretion management [28]. In proof-of-concept neuroimaging studies in healthy subjects, PES modulated the organization of the cortical swallowing network [9, 29] and led to increased sensorimotor activation during swallowing after pharyngeal anesthesia [30]. In addition, PES was shown to increase substance P level in saliva in tracheotomized stroke patients, which was a predictor of decannulation success [31]. This suggests that in addition to the central triggering of neuroplasticity, peripheral sensory mechanisms may also contribute to the therapeutic effect of PES. Furthermore, pharmaceutical sensory stimulants such as capsaicin are also used to trigger substance P release. In two recent randomized controlled trials in PSD patients, swallowing function evaluated by a water swallow test was improved [32, 33]. In addition, data from another randomized trial with a cross-over design suggest that capsaicin leads to a short-term increase in motor cortex excitability in stroke patients [23]. This again highlights the complex central and peripheral interactions in the sensory system. In contrast, a study in healthy participants suggested that capsaicin has no central modulatory effect on the swallowing network [34].

In addition to the association between PSD and sensory impairment in general, this study also compared two different assessment methods of pharyngeal hypesthesia. Our results indicate, that in principle both the touch-technique and the FEES-LSR-Test are suitable for the detection of pharyngeal hypesthesia. The advantage of the LSR approach when used in studies is that a metric result is obtained, allowing for more sophisticated analyses than with a binary or ordinal result as in the touch-technique. As in the validation study with healthy subjects [18], our study also showed a decrease in LSR with increasing trigger volume, however as expected with longer LSR compared to the healthy subjects. This shows that an increased sensory stimulus (larger trigger volume) is associated with a decreased LSR and thus suggests that LSR is a suitable marker of pharyngeal sensation in this patient population. When considering the different trigger volumes, the correlation with the touch-technique was found only with 0.3 ml and 0.4 ml trigger volumes. This suggests that pharyngeal hypesthesia in PSD can be quantified by LSR with these volumes, in contrast to the volumes of 0.2 ml and 0.5 ml. At 0.2 ml, the trigger may be too weak, so that a delayed reaction may occur even in the presence of clinically irrelevant hypesthesia. Conversely, at 0.5 ml, the trigger may be too strong so that a timely reaction may be elicited even in the presence of relevant sensory impairment. In the regression analyses, 0.4 mL showed an association with several parameters studied (severity of dysphagia, Murray secretion scale impairment, and with absent or delayed swallowing reflex). Therefore, 0.4 mL is particularly well suited to distinguish between the physiologic state and hypesthesia. Consistent with this, both the validation study and a study of healthy subjects over 70 years of age showed the largest effect size or differences in LSR at 0.4 ml to distinguish pharyngeal anesthesia and presbyphagia, respectively, from the physiologic state [3, 18]. This is further corroborated by a study in stroke patients, in which 0.4 ml trigger volume was superior to 2 ml in detecting aspirations using a swallow provocation test [35].

Another quantitative method that has been proposed to determine pharyngeal sensitivity is the so-called air-pulse method, or Flexible Endoscopic Evaluation of Swallowing with Sensory Testing (FEESST). Here, an air-pulse is applied to the anterior pharyngeal wall via a second endoscopic working channel and the pressure threshold of the air pulse is determined at which the laryngeal adductor reflex is triggered [36, 37]. However, the clinical relevance and reliability of this method is controversial. Some authors report that the results of the air-pulse method, unlike the touch-technique, cannot be associated with penetration and aspiration [12], or that the interobserver agreement is low [38]. One possible source of error is the difficulty in achieving a constant distance to the pharyngeal wall with the endoscope [12]. This problem is countered in swallow provocation tests by using different, easy-to-dose amounts as triggers (with the hypothesis that this makes the exact location of application less relevant). However, in comparison to the FEES-LSR-Test, the touch-technique in our study demonstrated to be equally valid in detecting deficits in pharyngeal sensitivity. Therefore, this method can be considered a useful and straightforward approach for clinical practice.

There are different limitations that must be considered when interpreting the results of this study. On the one hand, both test methods used rely on a motor response and cannot conclusively differentiate between an afferent and an efferent problem. Electrophysiological methods such as sensory evoked potentials are required for this purpose. When evaluating the results of the touch-technique the raters were not blinded to the FEES-results. Furthermore, individual regression analyses did not fulfill the condition of proportional odds according to the test of parallel lines, so that in these cases the results must be interpreted with caution. In addition, many patients who did not wish to participate or for whom it was not possible to inform their caregivers in time were not included in the study. Thus, a selection bias may have occurred and generalisability to other cohorts may be limited. In addition, the cross-sectional design and lack of follow-up do not allow conclusions about the temporal dynamics of reported outcomes.

## Conclusion

Pharyngeal hypesthesia is an independent predictor of PSD severity as well as impaired secretion management. PSD caused by sensory impairment is mediated by an absent or attenuated swallowing reflex. Other PSD pathologies such as pharyngeal residue or premature bolus spillage do not appear to be related to pharyngeal hypesthesia. Both, the swallow provocation test via the LSR as a quantitative method and the touch-technique with binary or ordinal results, respectively, are suitable as assessment methods of pharyngeal hypesthesia. In the former method, 0.3 ml or especially 0.4 ml is suitable as a trigger volume.

## Supplementary Information

**Additional file 1**: Table S1 Model fitting information of the regression analyses predicting increased Fiberoptic Endoscopic Dysphagia Severity Scale (FEDSS), increased Murray Secretion Scale, increased premature bolus spillage, increased pharyngeal residue and increased impairment of the swallowing reflex with the different sensory test procedures. LSR: Latency of Swallowing Response, df: degree of freedom.

## Data Availability

The disclosure of individualized and pseudonymized data to third parties not involved in the study is not possible according to the ethical vote. However, the authors can be contacted to ask for further anonymized data.

## Abbreviations

FEDSS: Fiberoptic Endoscopic Dysphagia Severity Scale
FEES: Flexible Endoscopic Evaluation of Swallowing
FEES-LSR-Test: Flexible Endoscopic Evaluation of Swallowing Latency of Swallowing Response Test
FEESST: Flexible Endoscopic Evaluation of Swallowing with Sensory Testing
IBM: International Business Machines Corporation
ICU: Intensive Care Unit
LSR: Latency of swallowing response
ml: Milliliter
mRS: Modified Rankin Scale
NIHSS: National Institute of Health Stroke Scale
PES: Pharyngeal electrical stimulation
PSD: Post-stroke dysphagia
SPSS: Statistical package for the social sciences
VFSS: Videoluoroscopic swallowing study
