# Supplementary material for: Relationship between post-stroke dysphagia and pharyngeal sensory impairment
Source: Neurol Res Pract. 2023 Feb 16;5:7. doi: 10.1186/s42466-023-00233-z (PMC9933330; doi:10.1186/s42466-023-00233-z)
Supplement: Supplementary file 1 — Additional file 1: Table S1 Model fitting information of the regression analyses predicting increased Fiberoptic Endoscopic Dysphagia Severity Scale (FEDSS), increased Murray Secretion Scale, increased premature bolus spillage, increased pharyngeal residue and increased impairment of the swallowing reflex with the different sensory test procedures. LSR: Latency of Swallowing Response, df: degree of freedom. [file 42466_2023_233_MOESM1_ESM.docx]

**Supplementary Table 1**

|  |  | **regression model** | | **test of parallel lines** | |
| --- | --- | --- | --- | --- | --- |
|  |  | χ2(df); Nagelkerke | p-value | interpretation | p-value |
| **FEDSS** | **LSR 0.3ml** | χ2(4)=20.6; 0.32 | **<0.001*** | violation | **<0.001*** |
|  | **LSR 0.4ml** | χ2(4)=21.9; 0.39 | **<0.001*** | fulfilled | 0.387 |
|  | **touch technique** | χ2(4)=30.0; 0.45 | **<0.001*** | fulfilled | 0.068 |
| **Murray** | **LSR 0.3ml** | χ2(4)=14.3; 0.25 | **0.006*** | fulfilled | 0.948 |
|  | **LSR 0.4ml** | χ2(4)=14.6; 0.30 | **0.006*** | fulfilled | 0.845 |
|  | **touch technique** | χ2(4)=20.0; 0.34 | **<0.001*** | fulfilled | 0.08 |
| **spillage** | **LSR 0.3ml** | χ2(4)=10.6; 0.20 | **0.032*** | fulfilled | 0.756 |
|  | **LSR 0.4ml** | χ2(4)=9.7; 0.22 | **0.045*** | fulfilled | 0.647 |
|  | **touch technique** | χ2(4) = 9.6; 0.19 | **0.047*** | fulfilled | 0.123 |
| **residue** | **LSR 0.3ml** | χ2(4)=3.3; 0.07 | 0.501 | n.a. | n.a. |
|  | **LSR 0.4ml** | χ2(4)=6.5; 0.15 | 0.163 | n.a. | n.a. |
|  | **touch technique** | χ2(4)=1.1; 0.03 | 0.89 | n.a. | n.a. |
| **reflex** | **LSR 0.3ml** | χ2(4)=18.3; 0.32 | **0.001*** | violation | **0.015*** |
|  | **LSR 0.4ml** | χ2(4)=28.7; 0.52 | **<0.001*** | violation | **0.019*** |
|  | **touch technique** | χ2(4)=18.8; 0.34 | **<0.001*** | fulfilled | 0.463 |

Supplementary Table 1: Model fitting information of the regression analyses predicting increased Fiberoptic Endoscopic Dysphagia Severity Scale (FEDSS), increased Murray Secretion Scale, increased premature bolus spillage, increased pharyngeal residue and increased impairment of the swallowing reflex with the different sensory test procedures. LSR: Latency of Swallowing Response, df: degree of freedom.
